# Health-related quality of life and influencing factors among migrant children in Shaoxing, China

**DOI:** 10.1186/s12955-017-0679-8

**Published:** 2017-08-30

**Authors:** Fengjiao Xu, Haiyan Xing, Wei Yu, Sanmei Chen, Hui Li

**Affiliations:** 1 Department of Nursing, School of Medicine, Shaoxing University, No.900 Chengnan Avenue Shaoxing, Zhejiang Province, 312000 China; 2 Institute of Epidemiology, Shaoxing Keqiao District Center for Disease Control and Prevention, Shaoxing city, Zhejiang province China

**Keywords:** Migrant children, HRQoL, PedsQL, Parental rearing style, Social support

## Abstract

**Background:**

Due to increasing export of labor service, many children following their parents leave from rural areas to urban areas in China. These migrant children might have psychological stress and lower quality of life. However, even up to this day, little is known about the health-related quality of life (HRQoL) of the migrant children. This study aims at investigating their living conditions and exploring the influencing factors of migrant children’s HRQoL.

**Methods:**

A cross-sectional survey of 856 migrant children, aged between 7 and 17, was conducted in Shaoxing. The 4 PedsQL 4.0 Generic Core Scales (Physical, Emotional, Social, School) were administered to reveal migrant children’s quality of life, while demographic data questionnaire, Egna Minnen av. Barndoms Uppfostran and Social Support Rating Scale were used to reflect the influencing factors.

**Results:**

For 824 effective questionnaires(all items were completed without any inconsistency in a questionnaire and all the information in the questionnaire is believable), the average age of these children was 12.80 ± 1.91.The average years that they stayed in Shaoxing were 6.41 years. The average score of HRQoL was 81.13 ± 10.77, Physical Functioning was 84.83 ± 12.49, Emotional Functioning was 71.32 ± 18.34, Social Functioning was 86.28 ± 14.12, and School Functioning was79.28 ± 13.16. There was no obvious difference (F = 0.138, *P* = 0.711) between boys and girls as for PedsQL. The score of PedsQL did not show significant association with migrant children’s gender and their school records, while school grade, the relationships with classmates, parental rearing style and social support showed significant correlations. Linear regression analysis showed that mother’s rejection, subjective support, father’s rejection, relationships with classmates, mother’s overprotection and level of using social support were influencing factors on PedsQL of migrant children.

**Conclusions:**

Migrant children scored lower on health-related quality of life, which was associated with parental rejection, mother’s overprotection, less subjective support, badly getting along with classmates and that they cannot use social support well.

## Background

With the rapid development of Chinese urban construction, more and more farmers leave their hometowns to the cities, hoping to find better employment chances. Some of them even bring their families to the cities, which is the typical characteristics of population mobility in recent years. As many children move to cities along with their parents, they constitute a new group called “migrant children”. According to China’s sixth population census in 2010 [1], migrant children aged 0-17 years amounted to 28.77 millions. Approximate 12 million migrant children were receiving compulsory education. These migrant children moved from rural area, which was completely different from urban area. During the process of adaptation to city life, their behaviors and inner thoughts have experienced a rise-and-fall period [2]. Admittedly, most migrant children’s parents engage in the relatively low-level manual labor, because their educational level is low and they have not received professional training [2, 3]. Therefore, they may have trouble in living within their means, and they have to work for such a long time that they do not have ample time to keep their children company, which may result in negative impact on migrant children. In fact, there are multiple studies focusing on migrant children, such as discussions about the receiving education rights, medical health (special health immunization), and the distinction between migrant children and local children [4–6]. Since 2000, many researchers have turned their attention to psychological status. They found that migrant children tended to have lower self-confidence [7], more anxieties, loneliness, depression and other emotional problems [8]. Nevertheless, few studies have revealed the comprehensive states of migrant children and how it took place. In order to have a better understanding of the living states of migrant children, in this study, we take advantage of HRQoL to investigate their conditions and try to find the influencing factors.

Health-Related Quality of life (HRQoL) is a multidimensional concept that includes physical, emotional and social health dimensions as delineated by the WHO [9, 10]. As far as adult was concerned, their HRQoL research has made significant progress. However, the pediatric quality of life research started quite late in China because lack of ideal measuring instrument [11]. But in fact, measuring children’s quality of life can comprehensively reflect the environment where the children grow up, which was extremely important for promoting children’s subjective well-being, life satisfaction and the establishment of social support [12]. As the proportion of the migrant populations is increasing in worldwide [13], and there is little data extending finds to schoolchildren and adolescents, it is crucial to measure HRQoL in migrant children [10]. And, it is significant to discover the factors which can impact the HRQoL of migrant children.

## Methods

A cross-sectional survey was carried out in 2014 in one district of Shaoxing. We chose to study migrant children now living in Zhejiang Province, which is one of provinces that have most migrant people in China. As many migrant children study in migrant schools which are constructed for migrant children, we used cluster sampling, randomly chose two migrant schools from 12 schools, including an elementary school and a junior school. All students participated in this questionnaire survey studying in above two schools from grade four to grade nine, so they can understand the questions and complete the survey all by themselves. One of our team members and a teacher from their school together administered the questionnaires from one class to another. In this study, migrant children had lived in Shaoxing at least 6 months, while most of them had stayed there more than 6 years. This study was approved by the ethics committee of our university, and all participants provided written informed consent before they completed questionnaire.

## Measures

### Demographic data

The following demographic variables were assessed in a self-report questionnaire including ages, grades, gender, years of migrant, their marks in class, and how they got along with their classmates.

### Pediatric quality of life inventory ™ 4.0 (PedsQL)

The PedsQL version 4.0 Generic Core Scales has 23 items, can be grouped into 4 domains of HRQoL: 1. Physical Functioning (8 items), 2. Emotional Functioning (5 items), 3. Social Functioning (5 items) and 4. School Functioning (5 items). These scales are feasible for child self-report including ages from 5 to 7, 8 to 12 and 13 to 18. Parent proxy-report includes ages from 2 to 4, 5 to 7, 8 to 12 and 13 to 18, and assesses parent’s perceptions of their children’s HRQoL [14]. In essence, items of each part are the same, which were just different in language expressions. Each item in the scale is about the frequency of something happened in recent 1 month. And score of each item can be divided into five grades(0 ~ 4). When calculating, the scores need corresponding translation into 100 ~ 0. Scores of every aspect come from the sum of its subordinate items scores divided by the number of its subordinate items, and score of the whole scale is the sum of each item score divided by the number of whole scale [15]. Total score and each aspect score are between 0 ~ 100, meanwhile, the higher score means the child live with better quality of life. In this study, we selected self-report PedsQL4.0 Chinese version [11] as instrument, which had be confirmed having great validity and reliability [16] and was calculated for Chinese children’s quality of life study.

### Egna Minnen av. Barndoms Uppfostran(EMBU)

In order to evaluate parental rearing styles easier, Arrindell [17] extracted 46 items from standard Egna Minnen av. Barndoms Uppfostran, according to the subject content and psychometrics indexes, as a result, formed Short-Egna Minnen av. Barndoms Uppfostran(s-EMBU).s-EMBU is self-report questionnaire, including father’s and mother’s version which has the same subject and content and has 23 items respectively, and all items score from 1 ~ 4. It contains three sub-dimensions: Rejection, Emotional Warmth and Over Protection. The coefficient of internal consistency of the scale is among 0.74 ~ 0.84, test-retest reliability is among 0.70 ~ 0.81 [18].

### Social support rating scale(SSRS) [19]

Social support was assessed by the SSRS. The SSRS is a ten-item scale with three subscales which respectively are subjective support (4 items), objective support (3 items) and the level of using social support (3 items). The higher score of the whole scale and each subscale indicate better social support. SSRS has been widely used to evaluate social support because it is simple and convenient, easy to understand, and has good validity and reliability. Previous research have demonstrated [20] test-retest reliability of SSRS was 0.92 and intrinsic consistency of each item was among 0.89 ~ 0.94.

### Data analysis

The SPSS 19.0 was used for statistical analyses. One-Way ANOVA analyses and Nonparametric Tests were used to find the relations between demographic data and the total score of PedsQL version 4.0 Generic Core Scales. Linear Correlation analyses were used to explore the connections among Social Support, Parent Rearing Styles and the scores of migrant children HRQoL. Linear regression analyses were used to predict the factors that influence the migrant children’s quality of life.

## Results

### Participants characteristic

Descriptive statistics on study variables are presented in Table 1. There were 856 migrant children participated in this study, while we acquired 824 effective questionnaires (all items were completed and without any inconsistency in a questionnaire), and effective return ratio was 96.26%. Because all the questionnaires were completed anonymously, we cannot know exact reasons why these 32 students submitted futile questionnaire. Maybe the questionnaire including too many items for a few students, or the students fail to understand correctly some questions in the questionnaire. There were 481 boys accounting for 58.4% of the total number and 343 girls accounting for 41.6% of the total number completed the survey. The minimum age of the respondents was 9 and the maximum age was 17, and an average age was 12.80 ± 1.91 years. The average years that they stayed in Shaoxing were 6.41 years. More than half (64.2%) migrant children thought they got along with their classmates well, while minority (2.1%) children could not get along well with others. For all participants, the average score of HRQoL was 81.13 ± 10.77, Physical Functioning was 84.83 ± 12.49, Emotional Functioning was 71.32 ± 18.34, Social Functioning was 86.28 ± 14.12, and School Functioning was79.28 ± 13.16.

**Table 1:** demographics of migrant children

	Group	*N*	%
Gender	Boy	481	58.4
	Girl	343	41.6
School grade	4th grade	150	18.2
	5th grade	100	12.1
	6th grade	104	12.6
	7th grade	178	21.6
	8th grade	188	22.8
	9th grade	104	12.6
School record	Poor	235	28.5
	General	317	38.5
	Good	272	33.0
Relations with classmates	Bad	17	2.1
	Normal	278	33.7
	Well	529	64.2

### Determinants of PedsQL

Table 2 revealed that there was no obvious differences (F = 0.138, *P* = 0.711) between boys and girls as for PedsQL. Meanwhile, the score of PedsQL did not show significant association with migrant children’s gender, and the same result found in their school records. As far as school grades and relationships between students were concerned, we used Nonparametric Tests as their heteroschedasticity. The analysis results were shown in Table 3. Migrant children in different grades got significant distinctions as for PedsQL(P<0.01). When they got along well with classmates, they are more likely to have higher PedsQL scores. Linear Correlation analyses results were summarized in Table 4. The migrant children lived with their parents with different rearing patterns would be related to their quality of life, and PedsQL score also changed with the subjective and objective support migrant children obtained. The children who can utilize social support would live with higher quality of life.

**Table 2:** variance analysis of gender, school record and PedsQL

Variable	Group	PedsQL $\left(\overline{\mathrm{X}}\pm \mathrm{S}\right)$	*F*	*P*
Gender	Boy	81.24 ± 10.84	0.138	0.711
	Girl	80.96 ± 10.69		
School record	Poor	81.28 ± 11.19	0.619	0.539
	General	81.51 ± 10.39		
	Good	80.55 ± 10.86

**Table 3:** Kruskal-Wallis Test between school grade, relations with classmates and PedsQL

Variable	Group	*N*	Average rank	*χ* ^2^	*P*
School grade	4th grade	150	403.85	15.287	<0.001
	5th grade	100	397.02		
	6th grade	104	455.03		
	7th grade	178	388.90		
	8th grade	188	452.84		
	9th grade	104	364.79		
Relations with classmates	Bad	17	226.85	31.723	<0.001
	Normal	278	363.28		
	Well	529	444.33

**Table 4:** Correlations about PedsQL with parental rejection, parental emotional warmth, parental overprotection, objective support, subjective support and using of support

	Q	FR	MR	FEW	MEW	FO	MO	OS	SS	UOS
Q	1									
FR	-0.28**	1								
MR	-0.28**	0.74**	1							
FEW	0.15**	−0.27**	−0.24**	1						
MEW	0.18**	−0.19**	−0.31**	0.75**	1					
FO	-0.12**	0.41**	0.25**	0.13**	0.16**	1				
MO	-0.13**	0.36**	0.36**	0.07	0.18**	0.78**	1			
OS	0.30**	−0.21**	−0.25**	0.51**	0.46**	0.03	0.02	1		
SS	0.13**	−0.14**	−0.16**	0.31**	0.27**	0.02	0.01	0.69*	1	
US	0.18**	−0.16**	−0.16**	0.32**	0.31**	−0.04	−0.05	0.34**	0.34**	1

*Q* PedsQL, *FR* father rejection, *MR* mother rejection, *FEW* father’s emotional warmth, *MEW* emotional warmth, *FO* father’s overprotection, *MO* mother’s overprotection, *OS* objective support, *SS* subjective support, *US* using of support

** indicate *P*<0.001,* indicate *P*<0.05

### Linear regression analyses results

We chose variables which were related to migrant children PedsQL score as independent variables, and set total PedsQL score as dependent variables to do linear regression analyses, tried to explore the factors that affected migrant children quality of life in this study. The result demonstrated that mother’s rejection, subjective support, father’s rejection, and relationships with their classmates, mother’s overprotection and level of using social support were predicted variables for PedsQL. It means the children who receive less subjective support, who cannot make good use of the support they get, who get badly along with their classmates in school, whose parents deny and reject them, whose mother protect them too much, live with a lower quality of life (Table 5).

**Table 5:** Stepwise Regression of PedsQL and influencing factors

variable	β	t	*p*	*R* ^*2*^	*F*	*P*
MR	-3.584	−2.606	0.009	0.28	33.355	<0.001
SS	0.469	4.456	0.000			
FR	-4.798	−3.458	0.001			
RWC	2.728	2.810	0.005			
MO	-2.732	−2.789	0.005			
US	0.498	2.160	0.031			

*RWC* relations with classmates

## Discussion

### Parental rearing styles & HRQoL

Health related quality of life is a concept which represents an individual’s own perception of his or her health status, functioning, and well-being in the domains of physical, psychological, social and role performance [21, 22]. It is a kind of multidimensional structure can exactly epitomise the human body health level [23], which was compromised by subjective satisfaction and objective state of lives. Consequently, something affecting migrant children’s emotional experiences and feelings can also affect their quality of life. In this study, parental rearing style significantly affected migrant children’s quality of life. According to regression analysis, we found that parents’ rejection and mother’s overprotection had a negative influence on migrant children’s quality of life. Parents’ rejection means parents are strict with the children, and punish the children more often [24]. A mother who overprotects child means a mother interferes in children’s life too much so that the children have no freedom [24]. And this kind of mother always tend to be over anxious and ignore the children’s demands. Virtually, the way of parental teaching was an important factor in the family education, and it had extensive, profound and lasting influence on the psychological development of migrant children [25]. In other words, parental rearing styles have a significant impact on children’s personality formation [26] and psychological health [27]. Children living with parents who always give them disadvantageous comments will be diffident, which challenges the children to adapt to school and establish a good relationship with classmates. Moreover, a child lacking self-confidence may be afraid to express himself in class, or even be ashamed of asking teacher questions. Therefore, getting good records in school becomes more difficult for him. This result is consistent with a study conducted in Iran, which found that children whose parents behave with authority display higher levels of adaption, psychological maturity, psychosocial efficiency, self-confidence and educational success, while children of authoritarian parents exhibit weak social skills and low self-confidence [28]. However, most migrant workers’ cultural degree is generally low and they have less opportunity to contact scientific knowledge of family education. Therefore, migrant parents showed more severe punishment and rejection to their children [29]. This study may corroborate that emotional warmth and understanding will have positive influence in migrant psychological health among educated migrant children. As a result, these migrant children will become more self-confident and have more positive emotional experiences in their lives. Thus, these children will live a higher quality of life. On the contrary, the fact that parents over interfere with or protect children, reject children’s reasonable request, deny children’s development and progress or fuss about children’s fault will have adverse impact on children’s mental health. Then, these kinds of improper parental rearing styles will lead to low quality of life of migrant children. As far as mother or father was concerned, our findings showed that mother’s rearing style have more influence on migrant children’ HRQoL level. In Chinese traditional culture [30], mother always takes more responsibilities than father for educating children in a family, because father needs to go out to work and feed his family.

### Social support & HRQoL

Social support [31] includes objective support or actual support which was visible (such as direct material assistance and social network), and subjective support or experience emotional support (such as satisfaction, the experience to be understood and respected). Objective support which can be either physical or specific and direct aid services also can be advices or guidance or persuasions, that help to solve the problems, can also be a sense of social belonging through providing social networks [32]. Hu XY [33] found that the higher objective support the migrant children get, the more resource they would acquire from social networks. These resources can be effective buffer adverse factors in the lives of migrant children, and promote their life satisfaction and mental health. Emotional support refers to providing empathy, attention, emotion, acceptance and trust, encourage etc. Actually, social support not only affords economical and material support, which can immediately promote migrant children’s quality of life. But also, social support can provide support about emotion and mentality that can perfect children’s psychosocial condition [32]. Nowadays, social support of available external resources has got increasingly concerned in the field of psychosomatic medicine. In this study, we tested if the observed decrease in HRQoL among migrant children was mediated by social support. Previous researched showed that great social support can improve patients’ HRQoL level [34], great social support and (or) good usage of social support can promote the migrant children’s social adaptation, mental and physical health [35]. Our study also convinced that social support has positive relationship with migrant children HRQoL level, subjective support and utilizing social support are independent prediction factors for HRQoL. The children with efficient usage of support and perceiving more support will buffer the negative influence of stress in their lives, migrant children will achieve their own development and socialization process through constant interaction with social support main body. Moreover, higher subjective support scores means the children can perceive more support in reality, undoubtedly, these children would be willing to actively seek help. Therefore, social support can boost migrant children quality of life by affecting their mental health and social adaptiveness.

### Relationship with classmates & HRQoL

Our findings showed that apart from parent’ rearing styles and social support, how migrant children get along with their classmate also impresses function on migrant children’s HRQoL. To our knowledge, migrant children getting along well with their classmates may mean they adapt to school life well, they have more friends in school so they can get more social support compared with those who get along badly with their classmates. As school adaptation and social functioning are inherent parts of PedsQL, it is no accident that the children who get along in class well would have higher quality of life.

### Limitations

Some limitations of the study deserve comment. Our results are based on a sample, however, all our research objects came from Shaoxing. In order to acquire more deep-going evidence we should expand sampling frame in future research. As far as we know, there exist numerous factors which may influence the migrant children’s HRQoL. However, in our study, we only explored the external factors, while some interior causes(as personality, disposition, habitus et al.) can also lead to migrant children’s HRQoL transformation.

## Conclusion

HRQoL of migrant children is related with parental rejection, mother’s overprotection, social support and how they get along with their classmates. Parental rearing style should be considered as one of the numerous potential determinant factors that contribute to migrant children’s quality of life. Positive rearing style such as emotional warmth should be encouraged, while rejection and overprotection are negative styles and should be abandoned in order to facilitate HRQoL of migrant children in China. In daily life, the parents of migrant children should evaluate and encourage their children in a more positive way, and respect their children’s opinions when they have disagreements. Furthermore, getting more subjective support, using social support well and getting along well with classmates will accelerate migrant children’s HRQoL. Parents and teachers as important social support resources should pay more attention to migrant children and afford support that migrant children can realize. Teachers can also encourage migrant children to be more confident and make more friends in school. Finally, migrant children as a big social group with many negative experiences, our whole society should concern for their lives and promote them growing up healthily. Based on our findings, we appeal to the relative department to attach importance to the problem of migrant workers and migrant children. Firstly, media can strengthen advocation to assist migrant children families and eliminate discrimination against migrant children family from local residents. Secondly, local government may build registration and management system of the migrant population and regularly interview these families. When some children are down and out, encounter domestic violence, addictive drugs in the wrong direction, government should provide positive assistance and support for them. Thirdly, in the aspect of education, Education Department may establish equal admission policy insure that every migrant child can receive nine-year compulsory education. Then, caring for migrant children growing-up health, school should play a leading role and focus on migrant children’s psychological health and life needs rather than school record only. Finally, as for health care, relative government expand health insurance coverage and ensure migrant children access to basic medical and health services easily. Exactly, if more departments work together, migrant children will live in a higher quality of life and grow up healthily.

## Abbreviations

EMBU: Egna Minnen av Barndoms Uppfostran
HRQoL: Health-Related Quality of Life
PedsQL: Pediatric Quality of Life Inventory
SSRS: Social Support Rating Scale
